# ABHD11 Is Critical for Embryonic Stem Cell Expansion, Differentiation and Lipid Metabolic Homeostasis

**DOI:** 10.3389/fcell.2020.00570

**Published:** 2020-07-07

**Authors:** Gaoke Liu, Yan Ruan, Junlei Zhang, Xueyue Wang, Wei Wu, Ping He, Jiali Wang, Jiaxiang Xiong, Yuda Cheng, Lianlian Liu, Yi Yang, Yanping Tian, Rui Jian

**Affiliations:** ^1^Laboratory of Stem Cell and Developmental Biology, Department of Histology and Embryology, College of Basic Medical Sciences, Third Military Medical University, Chongqing, China; ^2^Department of Thoracic Surgery, Southwest Hospital, First Affiliated Hospital Third Military Medical University, Chongqing, China; ^3^Cardiac Surgery Department, Southwest Hospital, First Affiliated Hospital Third Military Medical University, Chongqing, China; ^4^Experimental Center of Basic Medicine, College of Basic Medical Sciences, Third Military Medical University, Chongqing, China

**Keywords:** ABHD11, embryonic stem cell, self-renewal, overexpression, knockout

## Abstract

Growing evidence supports the notion that lipid metabolism is critical for embryonic stem cell (ESC) maintenance. Recently, α/β-hydrolase domain-containing (ABHD) proteins have emerged as novel pivotal regulators in lipid synthesis or degradation while their functions in ESCs have not been investigated. In this study, we revealed the role of ABHD11 in ESC function using classical loss and gain of function experiments. Knockout of *Abhd11* hampered ESC expansion and differentiation, triggering the autophagic flux and apoptosis. In contrast, *Abhd11* overexpression exerted anti-apoptotic effects in ESCs. Moreover, *Abhd11* knockout disturbed GSK3β/β-Catenin and ERK signaling transduction. Finally, *Abhd11* knockout led to the misexpression of key metabolic enzymes related to lipid synthesis, glycolysis, and amino acid metabolism, and ABHD11 contributed to the homeostasis of lipid metabolism. These findings provide new insights into the broad role of ABHD proteins and highlight the significance of regulators of lipid metabolism in the control of stem cell function.

## Introduction

Embryonic stem cells (ESCs) are derived from the inner cell mass of early preimplantation embryo and can proliferate unlimitedly while maintaining the potential to differentiate into all somatic lineages (Nichols and Smith, 2011). Due to their self-renewal ability and pluripotency, ESCs represent a cell model for recapitulating and investigating the developmental processes, and offer unique opportunities in regenerative medicine (Wu and Belmonte, 2016). Over the past two decades, research efforts focusing on ESC biology have led to identify an orchestrated regulation involving epigenetic, transcriptional, and signaling networks, promoting pluripotency during self-renewal (Ng and Surani, 2011; Li et al., 2012; Hassani et al., 2014). Recently, growing evidences have shown that the metabolic state of ESCs is an emerging indicator of pluripotency and self-renewal (Zhang et al., 2012; Ryall et al., 2015; Sperber et al., 2015; Teslaa and Teitell, 2015). A molecular understanding of cellular metabolic homeostasis during self-renewal is essential for harnessing the full potential of ESCs.

It has been established that glucose and amino acid metabolism, along with transcriptional and epigenetic regulation, are critical for ESC pluripotency and reprogramming. For instance, glycolysis, which mediates changes in acetyl-CoA and histone acetylation, controls ESC proliferation, pluripotency, and differentiation (Kondoh et al., 2007; Moussaieff et al., 2015; Gu et al., 2016). Threonine and methionine metabolism regulates pluripotency by affecting histone methylation (Wang et al., 2009; Shyh-Chang et al., 2013; Shiraki et al., 2014). Nevertheless, the role of lipid metabolism in the regulation of self-renewal and pluripotency has been poorly investigated at the molecular level. Recent studies have revealed that the state of lipid metabolism is tightly related to the maintenance of ESC identity (Yanes et al., 2010; Zhang H. et al., 2016; Wang et al., 2017; Cornacchia et al., 2019). ESCs are characterized by the presence of abundant metabolites, including free fatty acids and secondary lipid messengers (Yanes et al., 2010). Moreover, both the lipid profile and the expression of enzymes responsible for lipid synthesis change significantly during the course of ESC differentiation, as well as during somatic cell reprogramming to pluripotency (Panopoulos et al., 2012; Wang et al., 2017). Furthermore, lipid supplements in culture medium, such as oleic acid (Wang et al., 2017) and albumin-associated lipids (Cornacchia et al., 2019) have been shown to regulate ESC pluripotency, proliferation or differentiation. However, although the lipogenic requirements for ESC pluripotency and proliferative behavior have recently been established, how the molecules implicated in lipid metabolism regulate ESC self-renewal and differentiation is still largely unclear.

Most recently, the alpha/beta hydrolase domain (ABHD) protein family members have emerged as novel pivotal regulators of lipid metabolism and signal transduction, playing roles in metabolic disease and cancers (reviewed in Lord et al., 2013). The ABHD protein family comprises more than 19 proteins, most of which have a conserved GXSXG lipase motif, predicting their possible role in lipid synthesis or degradation (reviewed in Lord et al., 2013). For instance, ABHD5 is a highly conserved regulator of lipolysis (Lass et al., 2006), while ABHD6, a monoacylglycerol hydrolase, is a critical regulator of *de novo* fatty acid synthesis (Thomas et al., 2013). In addition to their roles in lipid metabolism, ABHD proteins exhibit distinct functions in cell proliferation. For example, ABHD5 plays a critical role in the induction of autophagy and apoptosis (Peng et al., 2016), while ABHD2, a triacylglycerol lipase (M et al., 2016), promotes prostate cancer cell proliferation and migration (Obinata et al., 2016). However, although recent research has greatly improved our fundamental understanding of ABHD proteins in lipid metabolism and cell biology, the biochemical and physiological functions of the majority of these proteins in ESCs are still largely unknown.

In this study, we uncovered the existence of biological roles of ABHD11 in the maintenance of mouse ESCs. Our findings that ABHD11 functions as a key regulator in lipid metabolism and is also required for the expansion and differentiation of ESCs provide deeper insights into the involvement of lipid metabolism in the regulation of ESC function and differentiation.

## Materials and Methods

### Plasmids Construction and Transfection

CRISPR/Cas9 was applied for the knock-in of *tTR-KRAB* inserts into *Rosa26* of R1 ESCs (Chu et al., 2016). The donor vector (pDonor-R26-tTR-KRAB-2AN) was generated by inserting a cassette of *tetR-KRAB-2A-NeoR* into Ai9 (Addgene, #22799) vector. The sgRNA sequence (CAGTCTTTCTAGAAGATGGG) directing a cut at 1219 bp upstream of the *Rosa26* transcription start site was inserted into the CRISPR plasmid PX330 (Addgene, #42230). The *tetO-CAG-Abhd11-RFP-IRES-HygroR* cassette was cloned into the pPyCAGIP vector (a gift from Ian Chambers). The sgRNA sequence (TGTCTCCCAGCCAGATGTTG) targeting *Abhd11* was cloned into the pLentiGuide-Puro vector (Addgene, #52963) or the pLentiCRISPR v2 vector via *Bsm*BI restriction enzyme sites (a gift from Feng Zhang, Addgene, #52961). The pLenti-Cas9-Blast vector was obtained from Addgene (#52962). The GFP-LC3 plasmid was a gift from Dr. Hong Zheng. All plasmids and construction details are available on request. The plasmid DNA was transfected using Lipofectamine 2000 (Invitrogen) according to the manufacturer’s instructions.

### Cell Culture

R1 murine ESCs were maintained as previously described (Zhang et al., 2014). Briefly, ESCs were plated on 0.2% gelatin-coated plates in ESC maintenance medium containing high glucose Dulbecco-modified Eagle medium (DMEM), 5% fetal bovine serum (FBS), 15% KnockOut Serum Replacement (KSR), 2 mM Glutamax, 1 mM sodium pyruvate, 0.1 mM NEAA, 0.1 mM β-mercaptoethanol, 100 U/ml penicillin, 100 U/ml streptomycin (all from Invitrogen) and 10 ng/ml LIF (Millipore). Cells were routinely propagated by trypsinization and replated every 2 to 3 days, with a split ratio of 1:10. For ERK inhibition, 1 μM PD0325901 was added in the maintenance medium. For 2I/LIF condition culture, ESCs were split onto a gelatin-coated dish in DMEM/F12/N2B27 medium supplemented with 1 μM PD0325901, 3 μM CHIR99021, 10 ng/ml LIF, 0.1 mM β-mercaptoethanol, and 1 mg/ml BSA. Mouse embryonic fibroblasts (MEFs) were cultured in medium containing DMEM, 10% FBS, 2 mM Glutamax, 0.1 mM NEAA. All cell cultures were maintained at 37°C under 5% CO_2_.

### Cell Differentiation

For monolayer differentiation, ESCs were seeded at a density of 2 × 10^4^ cells/cm^2^ in gelatin-coated plates and cultured for 3 days in the maintenance medium without LIF. For EB formation, single-cell suspensions were plated at a density of 1 × 10^4^ cells/cm^2^ in ultra-low attachment dishes and cultured in DMEM containing 15% FBS, 2 mM glutamax, 1 mM sodium pyruvate, 0.1 mM NEAA, 0.1 mM β-mercaptoethanol. The medium was changed every 2 days. EBs were harvested at Day 0, Day 3, and Day 5.

### Cell Proliferation and Colony Formation Assay

For proliferation assay, cells were seeded at a density of 1 × 10^5^ cells/well in 12-well plates and passaged every 2 days. Viable cells were counted using Cell Counter (Countstar) and trypan blue. For colony formation assay, cells were seeded a density of 200 cells/well in 12-well plates and cultured for 5 days. Alkaline phosphatase activity was detected with a BCIP/NBT alkaline phosphatase detection kit as previously described (Zhang et al., 2014).

### Lentiviral Production and Infection

Briefly, 293FT cells were grown to 80% confluence in DMEM/10% FBS. Then, 5.56 μg Lentiviral vector, 4.17 μg pSPAX2 and 2.78 μg pMD2G were co-transfected into 293FT cells by calcium phosphate transfection. Twelve hours after transfection, the medium was changed with DMEM/5% FBS. Two day later, viral supernatant was collected and concentrated by ultracentrifugation at 70,000 *g* for 2 h. For lentivirus infection, cells were then plated at a density of 1 × 10^4^ cells in 24-well plates and viral along with polybrene (4 μg/ml; Sigma) were added. After 36 h, cells were trypsinized and replated at 1 × 10^4^ cells per gelatin-coated 60-mm dish, and cultured in ESC medium supplemented with 1 μg/ml puromycin (Invitrogen) for 3 days.

### Flow Cytometric Analysis

For cell cycle analysis, cells were washed twice with phosphate-buffered saline (PBS) and fixed in 70% ethanol at –20°C overnight. Then, the fixed cells were washed and incubated in PBS containing 50 μg/ml propidium iodide, 50 μg/ml RNase A, 0.2% Triton X-100, and 0.1 mM EDTA for 30 min on ice. For apoptosis analysis, cells were harvested and stained with Annexin V-APC and propidium iodide. Following staining, samples were analyzed using a flow cytometer (ACEA Novocyte).

### Teratoma Formation and Histological Analysis

All of the animal experiments were approved by the Animal Ethical and Experimental Committee of Third Military Medical University. Teratoma formation and histological analysis was performed as described previously (Zhang et al., 2014). Briefly, 8 × 10^5^ ESCs were injected into the posterior flanks of nude mice. The *Abhd11*^OE^ ESCs and *RFP*^OE^ ESCs injected mice were treated with 1 mg/ml doxycycline in their drinking water. After 4 weeks, tumors were collected and analyzed by hematoxylin-eosin staining.

### RNA Isolation, Reverse Transcription, and PCR

RNA Isolation, reverse transcription, PCR and real-time PCR were performed as previously described (Ruan et al., 2017). All primers used in this study are listed in Supplementary Table S4.

### Immunofluorescence and Western Blotting

Cells were fixed in 4% paraformaldehyde for 20 min at 4°C, and permeabilized with 0.1% Triton X-100 for 15 min, followed by blocking with 10% FBS/PBS for 30 min. Cells were stained with anti-β-Catenin (CST,1:200) for overnight at 4°C, followed by the rabbit IgG secondary antibodies (1:1000) and counterstained with Hoechst. Images were captured with a ZEISS 780 inverted confocal microscope. Western blotting was performed as previously described (Zhang et al., 2014). The antibodies used in this study are given in Supplementary Table S5.

### Transcriptome Analysis

RNA-Seq transcriptome analysis was performed at Genminix Informatics Ltd., Co. (Shanghai, China) using Illumina HiSeq × 10 sequencing platform. The read counts for each gene were calculated, and the expression values of each gene were normalized by R package “DESeq2”. The differentially expressed genes (DEGs) were selected by the DESeq2. A *p*-values < 0.05 and an absolute value of the log_2_FC ≥ 0.5 were used as a threshold for significance. Among the DEGs, the up-regulated genes with counts less than 10 in the experimental group were removed, and the down-regulated genes with counts less than 10 in the control group were removed. The DEGs are listed in Supplementary Table S2. Heatmaps were generated by Hierarchical Clustering in Cluster 3.0 and visualised using Java Treeview. The reactome and GO enrichment analysis on DEGs were performed using g:Profiler^1^ and visualized using Cytoscape and Enrichment Map as previously reported (Reimand et al., 2019). The raw data are in GEO accession number GSE142067.

### GFP-LC3 Detection

Cells were transfected with plasmid GFP-LC3 (a gift from Hong Zheng) and cultured on gelatin-coated glass slides. 36 h after transfection, cells were fixed with 4% paraformaldehyde for 20 min and counterstained with Hoechst. Images were captured with a ZEISS 780 inverted confocal microscope and analyzed using CellProfiler software (Klionsky et al., 2016).

### Untargeted Relative Quantitative Lipidomics Assay

For sample preparation and lipid extraction, 6 × 10^5^ cells were homogenized with 200 μL water and 240 μL methanol. Then 800 μL of methyl tert-butyl ether was added and the mixture was ultrasound 20 min at 4°C followed by sitting still for 30 min at room temperature. The solution was centrifuged at 14000 *g* for 15 min at 10°C and the upper organic solvent layer was obtained and dried under nitrogen. Lipid analysis by liquid chromatography-tandem mass spectrometry (LC-MS/MS) and data analyses were performed as instructions by Shanghai Applied Protein Technology. Briefly, reverse phase chromatography was selected for LC separation using CSH C18 column (1.7 μm, 2.1 × 100 mm, Waters). Mass spectra were acquired by Q-Exactive Plus in positive and negative mode, respectively. ESI parameters were optimized and preset for all measurements as follows: Source temperature, 300°C; Capillary Temp, 350°C, the ion spray voltage was set at 3000 V, S-Lens RF Level was set at 50% and the scan range of the instruments was set at m/z 200–1800. Lipid species were identified with LipidSearch software version 4.1 (Thermo Scientific^TM^). For data analysis, principal component analysis (PCA) and partial least-squares-discriminant analysis (PLS-DA) were performed. Fold change of the lipid content between *Abhd11* KO or OE and control cells was calculated. The significant different lipid species, which were showed by volcano plots, were determined based on the combination of fold change, variable influence on projection (VIP) values (obtained from PLS-DA) and *P*-values (Student’s *t*-test) on the raw data.

### Transmission Electron Microscopy

Cell samples were fixed using 2.5% glutaraldehyde in PBS at 4°C overnight, then washed twice with 0.1 M PBS and postfixed with 1% osmium tetroxide for 2 h at 4°C. Samples were dehydrated in increasing concentrations of acetone and double stained with uranyl acetate in 70% acetone at 4°C overnight. Samples were embedded, sectioned and examined using JEM-1400plus transmission electron microscopy.

### Statistics

Statistical analysis was performed using the Statistical Package for Social Science. The Student’s *t*-test was used to analyze the statistical differences. Data were presented as mean value ± SD, and *P* < 0.05 was considered to be statistically significant. Each experiment was performed at least three times.

## Results

### ABHD11 Association With ESCs Is Revealed by Bioinformatic Analysis

To investigate whether ABHD proteins were implicated in mouse ESC maintenance, we first analyzed ESC transcriptome data (Supplementary Table S1) and a published genome-scale CRISPR-Cas9 knockout ESC dataset, in which genes essential for ESC self-renewal are ranked (Tzelepis et al., 2016). Notably, we found that *Oct4* and *Nanog*, two core pluripotency genes (Loh et al., 2006), were expressed at high levels, and included in the top-1000 ranked genes. Among ABHD family genes, only *Abhd11* was in the top-2500 ranked genes and expressed at a relatively high level, while the other members of the family were out of the top-5000 ranked genes (Supplementary Figure S1A). The promoters of genes that are required to maintain ESCs in a non-differentiated state are usually co-occupied by a couple of core pluripotency transcription factors (PTFs) (Boyer et al., 2005; Loh et al., 2006; Kim, 2008). We next analyzed the target promoters of nine PTFs in ESCs (Kim, 2008). Among the involved 797 genes, *Abhd11* was the only ABHD family genes whose promoter was occupied by more than four factors (Supplementary Figure S1B). Furthermore, we analyzed the mRNA expression data of ABHD family genes in OCT4-positive (undifferentiated) and OCT4-negative (differentiated) cells derived from ESCs (Zhou et al., 2007). We found that *Abhd11* and *Abhd10* were downregulated, while most other ABHD family genes, such as *Abhd 1*, *3*, *5*, *6*, and *7* were upregulated in OCT4-negative cells (Supplementary Figure S1C). Collectively, these data suggested that ABHD11 was involved in the maintenance of ESC identity.

### Construction of ESC Lines With Conditional *Abhd11* Expression

**FIGURE 1:**
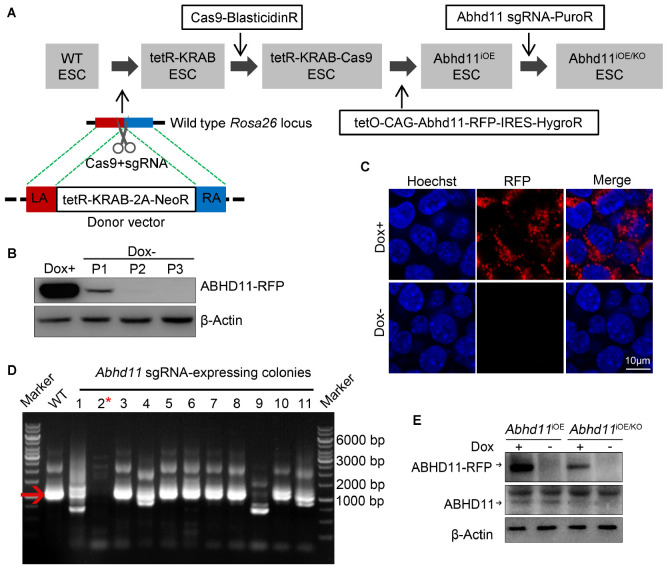
Generation and characterization of *Abhd11*^iOE^ ESCs and *Abhd11*^iOE/KO^ ESCs. **(A)** Schematic illustration of the four-step strategy for generating *Abhd11*^iOE^ ESCs and *Abhd11*^iOE/KO^ ESCs. Firstly, the *tetR-Krab* cassette was inserted into *Rosa26* locus. Secondly, lentivirus expressing Cas9 was transduced into tetR-KRAB-expressing ESCs followed by selection with blasticidin (2 μg/ml). Thirdly, the cells were transduced with the expression cassette of *tetO-CAG-Abhd11-RFP-IRES-HygroR* and selected with Hygromycin (200 μg/ml) and Dox (100 ng/ml). Finally, the *Abhd11*^iOE^ ESCs were infected with lentivirus expressing sgRNA against *Abhd11* and selected using puromycin (1 μg/ml). LA, left arm; RA, right arm. **(B)** Western blot analysis of the RFP expression in *Abhd11*^iOE^ ESCs cultured with Dox (1 μg/ml) or without Dox. Cells were subjected to serial passage (P) every 2 days. β-Actin was used as a loading control. **(C)** Fluorescence images of *Abhd11*^iOE^ ESCs cultured with Dox (1 μg/ml) or without Dox. After fixation, cells were counterstained with hoechst. **(D)** RT-PCR showing the expression of endogenous *Abhd11* mRNA in wild-type (WT) ESCs and *Abhd11* sgRNA-expressing colonies. The red arrow indicates the specific RT-PCR product (1002 bp) of the endogenous *Abhd11* mRNA. The red asterisk refers to a colony in which the endogenous *Abhd11* gene was knocked out. **(E)** Western blot analysis of ABHD11 using anti-ABHD11 antibody. *Abhd11*^iOE^ ESCs were cultured in the presence of 1 μg/ml Dox or without Dox. *Abhd11*^iOE/KO^ ESCs were cultured in the presence of 1 ng/ml Dox or without Dox. β-Actin was used as a loading control.

In order to assess the function of ABHD11 in ESCs, we attempted to generate *Abhd11* knockout (KO) and overexpression (OE) ESC lines. To circumvent the difficulty of obtaining *Abhd11*-null ESCs, which may have severe growth disadvantages, we attempted to firstly generate cells with inducible overexpression (iOE) of exogenous *Abhd11* using “tetR-KRAB-tetO” conditional gene expression system (Szulc et al., 2006) and then knock out the endogenous *Abhd11* using CRISPR/Cas9 technology (Cong et al., 2013; Mali et al., 2013). To this end, we generated a stable tetR-KRAB-Cas9-expressing cell line (Figure 1A), which was hereafter used as a control in all experiments. Next, we obtained *Abhd11*^iOE^ ESCs by transducing an inducible expression cassette of *Abhd11* targeted with Red fluorescent protein coding sequence (*Abhd11-RFP*), into the tetR-KRAB-Cas9-expressing cells (Supplementary Figure S2A and Figure 1A). The expression of *Abhd11-RFP* in subcloned *Abhd11*^iOE^ ESCs was induced in the presence of doxycycline (Dox) while silenced in the absence of Dox (Supplementary Figure S2A and Figures 1B,C). Finally, we generated *Abhd11*^iOE/KO^ ESCs by transducing a lentivirus carrying sgRNAs mapping to the endogenous *Abhd11* genomic sequence and spanning an intron-exon junction into *Abhd11*^iOE^ ESCs (Supplementary Figure S2B and Figure 1A). Expression of endogenous *Abhd11* in *Abhd11*^iOE/KO^ ESCs was detected by reverse transcriptase-PCR (RT-PCR), using specific primers targeting the untranslated region (UTR). One of the subclones failed to express normal levels of the endogenous *Abhd11* mRNA (Figure 1D). Western bot confirmed that, in the subclone, the endogenous ABHD11 protein was depleted, while exogenous ABHD11 could be efficiently switched on or off in 2 days (Figure 1E). Hence, endogenous *Abhd11*-depleted *Abhd11*^iOE/KO^ ESCs were used as *Abhd11*-KO (*Abhd11*^KO^) ESCs in the absence of Dox, and as *Abhd11*-rescued (*Abhd11*^RES^) ESCs in the presence of 1 ng/ml Dox; *Abhd11*^iOE^ ESCs were used as *Abhd11*-OE (*Abhd11*^OE^) ESCs in the presence of 1 μg/ml Dox.

### ABHD11 Contributes to Expansion of ESCs

**FIGURE 2:**
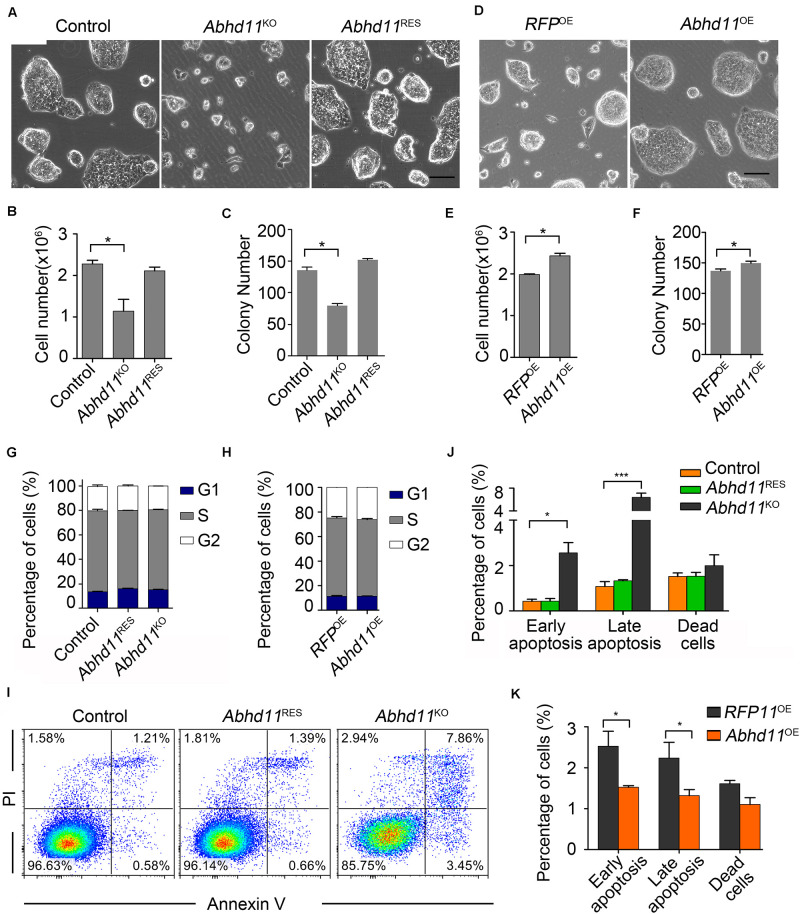
ABHD11 contributes to the expansion of ESCs. **(A)** Representative morphology of colonies formed by control, *Abhd11*^KO^, and *Abhd11*^RES^ ESCs. Scale bar, 100 μm. **(B)** Cell growth of control, *Abhd11*^KO^, and *Abhd11*^RES^ ESCs. **(C)** Quantitative analysis of colonies formed by control, *Abhd11*^KO^ and *Abhd11*^RES^ ESCs. **(D)** Representative morphology of colonies formed by the indicated cells. Scale bar, 100 μm. **(E)** Cell growth of *RFP*^OE^ ESCs and *Abhd11*^OE^ ESCs. **(F)** Quantitative analysis of colonies formed by *RFP*^OE^ and *Abhd11*^OE^ ESCs. **(B,E)** Cells (1 × 10^5^ cells/well in 12-well plates) were cultured for 2 days and cell numbers were counted. **(C,F)** Cells were plated at clonal density (200 cells/well in 12-well plates) and cultured for 5 days. **(G,H)** Proportions of the cells in G1, S and G2 phase. Cells were stained with propidium iodide (PI) and analyzed by fluorescence-activated cell sorting (FACS). **(I)** Apoptosis of the indicated cells was measured by FACS with Annexin-V and PI staining. One of three experiments with similar results was shown. **(J,K)** The fraction of the indicated cells labeled for early apoptosis (Annexin V^+^PI^–^), late apoptosis (Annexin V^+^PI^+^), and dead cells (Annexin V^–^PI^+^). Data in panels **(B,C,E–H,J,K)** were represented as mean ± s.d.; *n* = 3. *p < 0.05, ***p < 0.001.

To begin with, we characterized the phenotype of *Abhd11*-null ESCs. When plated in culture, *Abhd11*^KO^ ESCs formed smaller colonies (Figure 2A), and concurrently displayed a marked reduction in cell numbers compared to control cells (Figure 2B). Moreover, colony formation assays showed that the number of colonies formed by *Abhd11*^KO^ ESCs was less than the control cells (Figure 2C). Colony morphology, cell number, and colony-forming potential were restored in *Abhd11*^RES^ ESCs, and were indistinguishable from those of control cells (Figures 2A–C), indicating that the observed phenotypes in *Abhd11*^KO^ ESCs were specifically due to loss of ABHD11 function, rather than to possible off-target effects of CRISPR/Cas9 (Fu et al., 2013). However, in contrast to ESCs, MEFs expanded normally when infected with lentivirus expressing Cas9-sgRNA targeting *Abhd11* (Supplementary Figure S3), which suggested that the role of ABHD11 in the expansion of ESCs was cell context dependent. In contrast to *Abhd11*^KO^ ESCs, *Abhd11*^OE^ ESCs exhibited faster growth compared to *RFP* OE (*RFP*^OE^) ESCs (Figures 2D–F), indicating that *Abhd11* OE promoted ESC expansion.

### Opposite Effects of *Abhd11* KO and OE on Apoptosis

The opposite effects of *Abhd11* KO and OE on cell expansion led us to explore whether ABHD11 contributed to the regulation of the cell cycle or apoptosis. Cell cycle analysis showed that both *Abhd11*^KO^ and *Abhd11*^OE^ ESCs showed a normal cell cycle distribution (Figures 2G,H). However, we found that the number of apoptotic cells, as determined by Annexin V, significantly increased in *Abhd11*^KO^ ESCs (Figures 2I, 4J), suggesting that the loss of ABHD11 triggered apoptosis. In contrast, the fraction of apoptotic and necrotic cells among *Abhd11*^OE^ ESCs was approximately 50% of that of *RFP*^OE^ ESCs under different culture conditions, including maintenance, serum starvation, differentiation in the absence of leukemia inhibitory factor (LIF) or addition of retinoic acid (Figure 2K, and Supplementary Figure S4). These data showed that *Abhd11* OE exerted an anti-apoptotic effect in ESCs.

### *Abhd11* KO Induces Autophagy

**FIGURE 3:**
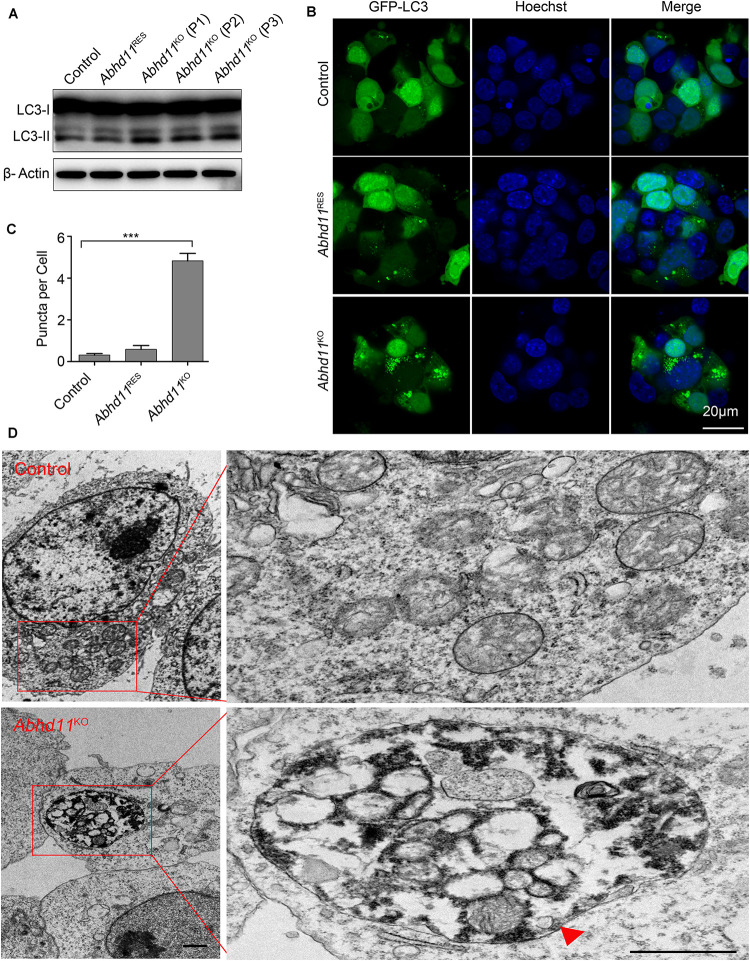
*Abhd11* KO induces autophagy. **(A)** Western blot analysis of LC3-I and LC3-II expression level in the indicated cells during passage (P) with anti- LC3 antibody. **(B)** Confocal fluorescence microscopy images showing the subcellular localization of GFP-LC3 in the indicated cells. **(C)** Quantification of GFP-LC3 puncta in the indicated cells. Data were represented as mean ± s.d. from three independent experiments and at least 50 cells in each experiment were counted. ***p < 0.001. **(D)** Representative TEM images of control ESCs and *Abhd11*^KO^ ESCs. A big autophagic vacuole appeared as double-membrane structure was highlighted with a red triangle. Scale bar, 1 μm.

It has been reported that autophagy (literally, ‘self-eating’), which is responsible for the maintenance of cellular homeostasis under metabolic stress conditions, plays a key role in the regulation of cell survival and death (Klionsky et al., 2016). We thus tried to address whether *Abhd11* KO in ESCs could induce autophagy. We found an increase in the amount of the active form of microtubule-associated protein light chain 3 (LC3-II), a marker of autophagy, in *Abhd11*^KO^ ESCs, compared to control ESCs (Figure 3A). We then transiently transfected cells with green fluorescent protein (GFP)-fused LC3 (GFP-LC3) to monitor the autophagic activity (Klionsky et al., 2016). Immunofluorescence confocal microscopy showed that GFP-LC3 was found to distribute evenly throughout the cytoplasm with few punctate spots in both control and *Abhd11*^RES^ cells. However, large typical GFP-LC3 dots, indicative of cytoplasmic autophagosomes, were observed in *Abhd11*^KO^ ESCs (Figures 3B,C). Transmission electron microscopy (TEM) detection also verified that large double-membrane autophagic vacuoles were present in *Abhd11*^KO^ ESCs (Figure 3D). In addition, when cultured in the presence chloroquine, which is a lysosomal inhibitor and prevents the degradation of LC3-II, both LC3II and large typical GFP-LC3 dots were increased in *Abhd11*^KO^ ESCs, compared to control cells (Supplementary Figure S5). Collectively, these results demonstrated that *Abhd11* depletion induced autophagy in ESCs.

**FIGURE 4:**
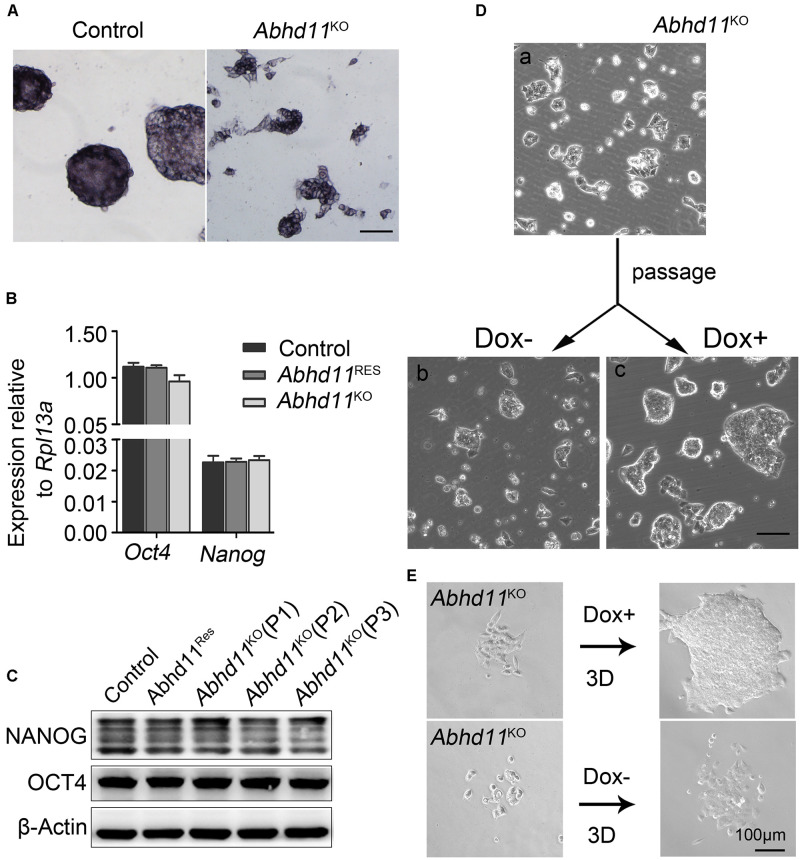
*Abhd11* is dispensable for the maintenance of pluripotency. **(A)** Alkaline phosphatase staining of colonies formed by the indicated cells. Scale bar, 100 μm. **(B)** qRT-PCR analysis of *Oct4* and *Nanog* mRNA level in the indicated cells. **(C)** Western blot analysis of OCT4 and NANOG expression level in the indicated cells during passage. **(D)** Representative morphology of colonies formed by *Abhd11*^KO^ ESCs at high colony density. **(E)** Morphologies of colonies formed by *Abhd11*^KO^ ESCs at low colony density.

### ABHD11 Is Dispensable for the Maintenance of Pluripotency

Next, we attempted to address whether *Abhd11* KO triggered ESCs to exit pluripotency and enter differentiation. We observed that the colonies formed by *Abhd11*^KO^ ESCs were still alkaline phosphatase-positive (Figure 4A), which was indicative of undifferentiated ESCs. In addition, *Abhd11*^KO^ ESCs displayed no changes in the expression of the pluripotency markers *Oct4* and *Nanog* (Figures 4B,C). Notably, *Abhd11*^KO^ ESCs could be maintained during serial passages (P) and rapidly recovered the ability to expansion and formed typical compact colonies at both high and low cell density when Dox was re-added to the medium (Figures 4D,E). These data indicated that *Abhd11*^KO^ ESCs maintained a pluripotent state without commitment to differentiation. In addition, we observed that *Abhd11*^OE^ ESCs also displayed no changes in alkaline phosphatase staining and Oct4 protein level compared to *RFP*^OE^ ESCs (Supplementary Figures S6A,B).

### ABHD11 Is Necessary for the Full Execution of Differentiation Programs

**FIGURE 5:**
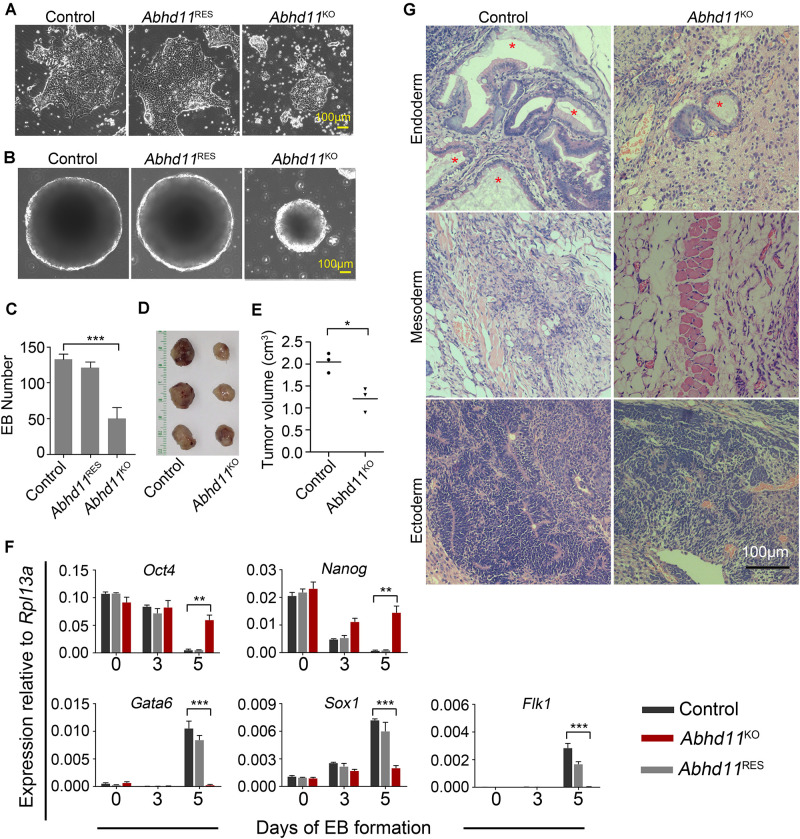
ABHD11 is necessary for the full execution of differentiation programs. **(A)** Representative images of the indicated cells cultured for 4 days in the absence of LIF. **(B)** Representative images of EBs formed by the indicated cells at day 5. **(C)** Quantification of EBs formed by the indicated cells at day 5. **(D)** Teratomas formed from the control and *Abhd11*^KO^ ESCs. Teratomas were collected 4 weeks after the injection of ESCs subcutaneously into nude mice. **(E)** The volume of teratomas presented in panel **(D)**. **(F)** qRT-PCR analysis of expression levels of pluripotency and germ layer marker during the course of EB formation. The EBs were collected at Day 0, 3, 5 during the course of EB formation. All data were normalized to *Rpl13a*. **(G)** Teratoma section were stained with hematoxylin/eosin. Asterisks indicate typical endodermal epithelium–like structures (simple columnar epithelium). Data in panels (**C**,**F)** were represented as means ± s.d.; *n* = 3. *p < 0.05, **p < 0.01, ***p < 0.001.

When plated on gelatin at clonal density and induced to differentiation in the absence of LIF, *Abhd11*^KO^ ESCs appeared smaller compared to the control cells (Figure 5A). In addition, the embryoid bodies (EBs) generated by *Abhd11*^KO^ ESCs were smaller and fewer than in control cells (Figures 5B,C). Furthermore, while both *Abhd11*^KO^ and control ESCs formed teratomas *in vivo*, the average size of teratomas formed by *Abhd11*^KO^ ESCs was significantly smaller compared to control ESCs (Figures 5D,E). To assess whether *Abhd11* deficiency affected the differentiation ability of ESCs *in vitro*, the expression patterns of pluripotency-associated genes and lineage-specific gene markers were subsequently assessed by quantitative reverse transcriptase-PCR (qRT-PCR). When induced to differentiate, *Abhd11*-KO cells failed to fully downregulate *Oct4* and *Nanog* (Figure 5F) upon cellular differentiation (Pesce and Scholer, 2001; Chambers et al., 2003). Moreover, under these conditions, *Abhd11*-KO cells did not normally induce the expression of early differentiation markers, such as *Gata6* (Endoderm), *Sox1* (Ectoderm), and *Flk1* (Mesoderm) (Figure 5F). In addition, although teratomas derived from *Abhd11*^KO^ ESCs contained cell types representing all three germ layers, *Abhd11*^KO^ ESCs derived little cells with vacuoles while control cells formed endodermal monolayer columnar epithelium consisting of enriched cells with vacuoles (Figure 5G). Taken together, these data demonstrated that ABHD11 was necessary for the full execution of differentiation programs. Moreover, ABHD11 OE could not block the differentiation of ESCs in the absence of LIF and *in vivo* (Supplementary Figures S6C–E).

### *Abhd11* KO Results in Disturbances in Signal Transduction

**FIGURE 6:**
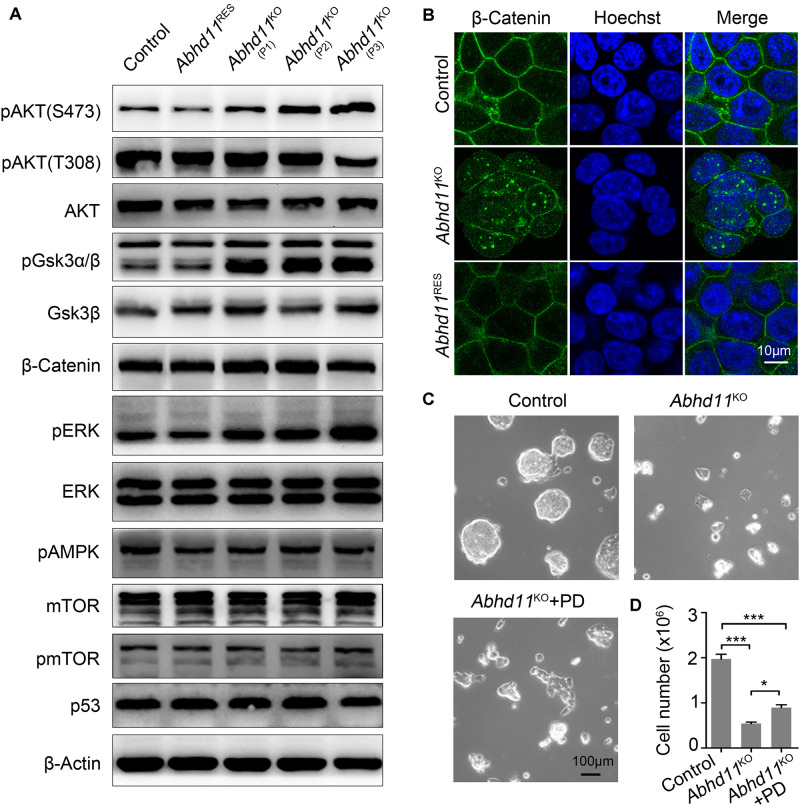
*Abhd11* KO results in disturbances in signal transduction. **(A)** Western blot analysis using antibodies as indicated. **(B)** Immunofluorescence analysis of β-Catenin in the indicated cells. Cells were counterstained with hoechst. **(C)** Representative morphology of colonies formed by control and P3 *Abhd11*^KO^ ESCs. *Abhd11*^KO^ ESCs were plated in duplicate and treated with 1 μM PD0325901 (*Abhd11*^KO^ + PD). **(D)** Cell numbers in panel **C** were counted. Data were represented as mean ± s.d. from three independent experiments. *p < 0.05, ***p < 0.001.

Recently, lipid metabolic intermediates have been demonstrated to participate in cellular signaling processes (Zechner et al., 2012). In light of the predicted role of ABHD11 in lipid metabolism (Arya et al., 2017), we then tried to identify which signaling pathways in ESCs could be affected by *Abhd11* depletion. It is well-known that ESCs can be maintained under defined culture conditions with the addition of LIF, which induces the activation of signal transducer and activator of transcription 3 (Stat3) and AKT (also known as protein kinase B, PKB) signaling (Niwa et al., 2009). We found that phosphorylated Stat3, total Stat3, and total AKT protein levels were not changed in *Abhd11*^KO^ ESCs (Figure 6A). However, there was an increase in AKT phosphorylation at Ser473, and a reduction of AKT phosphorylation at Thr308 in P3 *Abhd11*^KO^ ESCs (Figure 6A). This suggested a dysregulation of AKT activity in these cells. Nevertheless, the total level of AKT activity is difficult to assess, as it depends on the extent of phosphorylation at both Thr308 and Ser473 residues, as well as on its total protein concentration (Alessi et al., 1996).

Next, we found that, after Dox withdrawal, the extent of phosphorylation of glycogen synthase kinase 3 beta (GSK3β) (at Serine-9), was significantly increased in *Abhd11*^KO^ ESCs (Figure 6A). This suggested that GSK3β signaling was sensitive to the loss of ABHD11. It has been reported that Serine-9-phosphorylated GSK3β inhibits enzyme activity and leads to the nuclear accumulation of β-Catenin (Sato et al., 2004; Ding et al., 2005). Consistently, immunofluorescent staining showed an increase in β-Catenin translocation from the cytoplasm to the nucleus in *Abhd11*^KO^ ESCs (Figure 6B). These data indicated that *Abhd11* depletion had an impact on the regulation of the GSK3β/β-Catenin signaling pathway.

We also found that the extent of phosphorylation of the extracellular signal-regulated kinase (ERK) increased in *Abhd11*^KO^ ESCs (Figure 6A). Inhibition of ERK signaling enhances the self-renewal activity of ESCs (Burdon et al., 1999). To verify whether a higher extent of ERK phosphorylation could account for defective self-renewal in *Abhd11*^KO^ ESCs, we treated *Abhd11*^KO^ ESCs with the specific and widely used ERK phosphorylation inhibitor, PD0325901. We found that treatment with PD0325901 could not completely reverse the *Abhd1*1 KO-induced phenotype (Figures 6C,D), suggesting that additional factors were involved in the impairment of self-renewal observed in *Abhd11*^KO^ ESCs. The protein level of p53 and mTOR, as well as the phosphorylation of mTOR and pAMPK, were not altered in *Abhd11*-KO cells (Figure 6A). In addition, the detected signalings in *Abhd11*^OE^ ESCs showed no obvious changes (Supplementary Figure S6B).

### *Abhd11* KO Causes the Misexpression of Metabolic Genes

**FIGURE 7:**
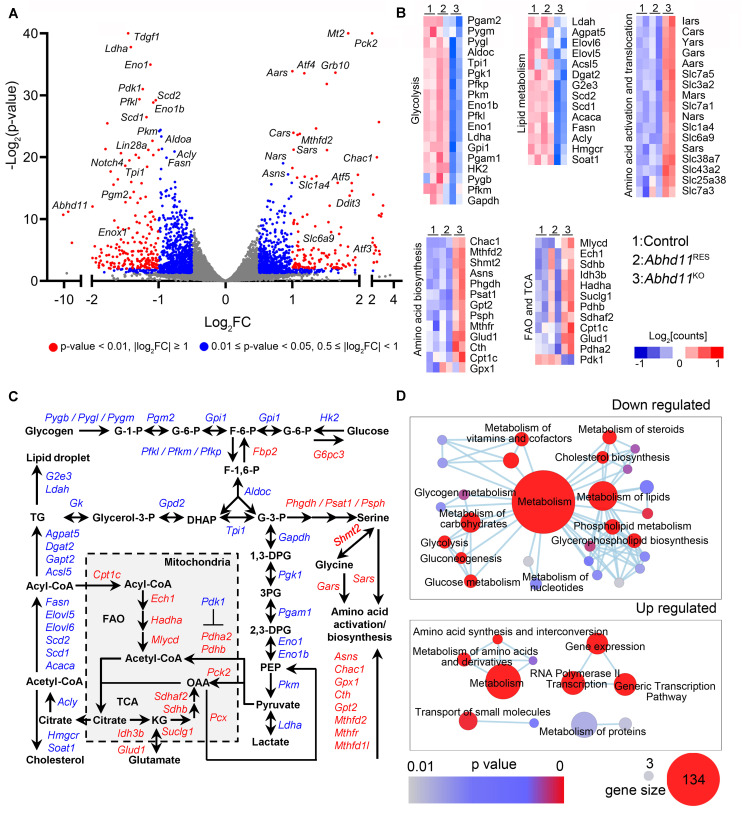
*Abhd11* KO causes the misexpression of metabolic genes. **(A)** Volcano plot of DEGs in P3 *Abhd11*^KO^ ESCs versus control ESCs. FC, fold change. **(B)** Heatmap of metabolic genes differentially expressed in *Abhd11*^KO^ ESCs versus control ESCs. **(C)** Biochemical pathways of genes that are up-regulated (red) and down-regulated (blue) as a result of *Abhd11* depletion. G-1-P, glucose 1-phosphate; G-6-P, glucose 6-phosphate; F-6-P, fructose 6-phosphate; F-1,6-P, fructose 1,6-biphosphate; G-3-P, glyceraldehyde 3-phosphate; DHAP, dihydroxy acetone phosphate; 1,3 DPG, 1,3-diphosphoglyceric acid; 3PG, 3-Phosphoglycerate; 2,3-DPG, 2,3-diphosphoglycerate; PEP, Phosphoenolpyruvate; OAA, oxaloacetate; FAO, fatty acid oxidation; TCA, citric acid cycle; KG, α-ketoglutarate; TG, triglyceride. **(D)** Enrichment map networks of reactome terms corresponding to down-regulated genes (upper panel, a part of the total networks that was presented in Supplementary Figure S7C) and up-regulated genes (lower panel) in P3 *Abhd11*^KO^ ESCs versus control ESCs. Reactome terms were visualized by the cytoscape plug-in: Enrichment Map. Node size represents the gene-set size and node color represents enrichment significance (as indicated). The deposited data can be found at: http://www.ncbi.nlm.nih.gov/geo/query/acc.cgi?acc=GSE142067.

To gain insights into the molecular mechanisms underlying ABHD11-dependent maintenance of self-renewal in ESCs, we characterized the transcriptome of *Abhd11*^KO^ ESCs by RNA seq. Notably, the Volcano Plot showed that the most significantly upregulated genes in *Abhd11*^KO^ ESCs, encoded for enzymes mainly involved in amino acid metabolism, such as *Aars*, *Nars*, *Sars*, *Cars*, and *Chac1* (Figure 7A). The most significant downregulated genes in *Abhd11*^KO^ ESCs included those for rate-limiting glycolytic enzymes (*pfkl*, *pkm*), lipid synthesis enzymes (*Scd1*, *Scd2*, *Fasn*, *Acly*), and *Lin28*, which plays a cooperative role in the regulation of pluripotency and metabolic proteome (Zhang J. et al., 2016; Figure 7A). Further analysis showed that *Abhd11* deletion caused a prominent upregulation of genes for amino acid activation, translocation, synthesis, fatty acid oxidation, and tricarboxylic acid cycle, and a substantial downregulation of key enzymes involved in lipid synthesis and of almost all glycolytic enzymes (Figures 7B,C, and Supplementary Table S2). Consistent with the above description, the expression of most of the pluripotency markers, such as *Oct4*, *Nanog*, *Sox2*, *Dax1*, *Klf4*, and so on, were not changed in *Abhd11*^KO^ ESCs (Supplementary Table S2).

Next, reactome in terms of the DEGs were analyzed and visualized by using Enrichment Map, an established Cytoscape plug-in (Reimand et al., 2019). In *Abhd11*^KO^ ESCs, the downregulated genes were functionally enriched in lipid and carbohydrate metabolism, as well as signal transduction, while the upregulated genes were enriched in amino acid metabolism and regulation of gene expression (Figure 7D and Supplementary Figure S7A). Consistently, gene ontology (GO) analyses of the DEGs, also revealed an enrichment in metabolic processes (Supplementary Table S3). Collectively, these data suggested that the loss of ABHD11 resulted in dysregulated expression of genes involved in multiple metabolic events.

Furthermore, we also analyzed DEGs between *Abhd11*^OE^ and control ESCs, and identified 261 downregulated and 216 upregulated genes in *Abhd11*^OE^ ESCs (Supplementary Figure S7B and Supplementary Table S2). The total number of deregulated genes in *Abhd11*^OE^ ESCs was much lower than in *Abhd11*^KO^ ESCs, suggesting a lower impact of *Abhd11* OE on overall transcription, compared to *Abhd11* KO. Notably, there was little overlap between DEGs in *Abhd11*^OE^ ESCs and DEGs in *Abhd11*^KO^ ESCs (Supplementary Figure S7C). In addition, the DEGs in *Abhd11*^OE^ ESCs were not enriched in metabolic genes and pluripotency related genes (Supplementary Table S2). These data suggested that *Abhd11* OE exerted little effects on the expression of metabolic genes.

### ABHD11 Contributes to the Homeostasis of Lipid Metabolism

**FIGURE 8:**
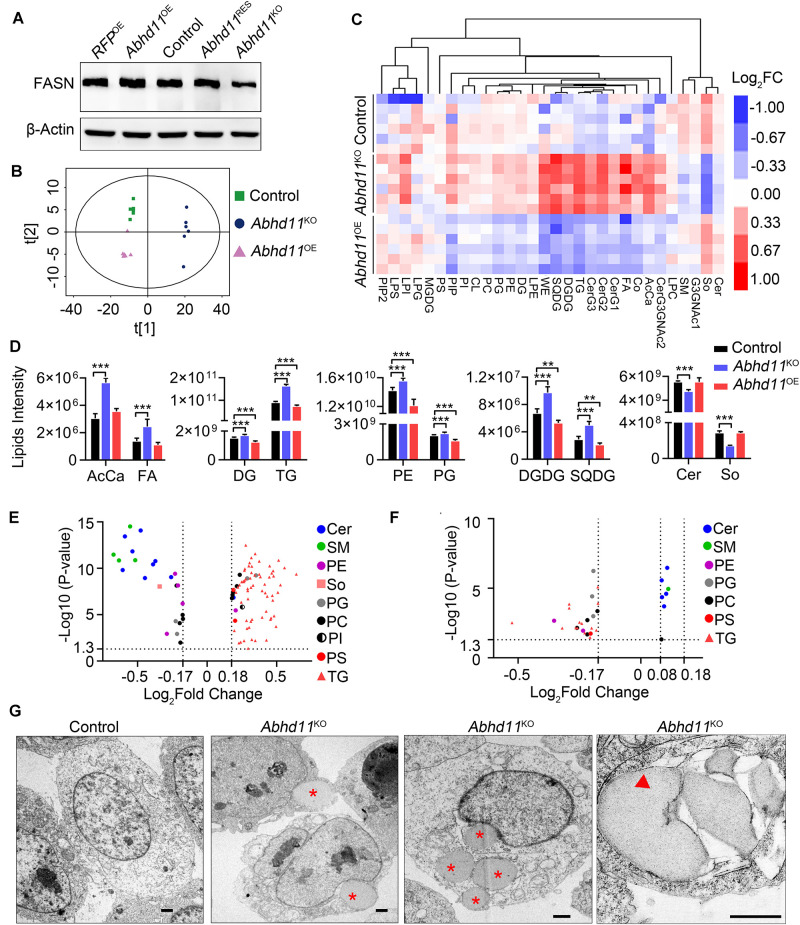
ABHD11 contributes to the homeostasis of lipid metabolism. **(A)** Western blot analysis of FASN expression level in the indicated cells. **(B)** Principal component analysis of lipid metabolites in control, *Abhd11*^KO^, and *Abhd11*^OE^ ESCs. **(C)** Heatmap of the 30 lipid classes content in control, *Abhd11*^KO^, and *Abhd11*^OE^ ESCs. AcCa, Acyl Carnitine; Cer, Ceramides; CL, Cardiolipin; Co, Coenzyme; DG, diglyceride; DGDG, Digalactosyldiacylglycerol; FA, Fatty acid; LPC, lysophosphatidylcholine; LPE, lysophosphatidylethanolamine; LPG, lysophosphatidylglycerol; LPI, lysophosphatidylinositol; MGDG, Monogalactosyldiacylglycerol; So, Sphingosine; SM, sphingomyelin; PC, phosphatidylcholine; PE, phosphatidylethanolamine; PG, phosphatidylglycerol; PI, phosphatidylinositol; PIP, phosphatidylinositol; PS, phosphatidylserine; TG, triglyceride; SQDG, Sulfoquinovosyldiacylglycerol; WE, wax exters. **(D)** Lipid intensity of lipid classes altered in *Abhd11*^KO^ and *Abhd11*^OE^ ESCs measured by LC-MS/MS. Data were represented as mean ± s.d. from six biologically independent samples. **p < 0.01, ***p < 0.001. **(E)** Volcano plot of lipid species that showed the most significant change in *Abhd11*^KO^ ESCs compared to control (fold change (FC) > 1.5 or FC < 0.67, *P*-value < 0.05, VIP > 1). **(F)** Volcano plot of lipid species that showed the most significant change in *Abhd11*^OE^ ESCs compared to control (FC > 1.2 or FC < 0.67, *P*-value < 0.05, VIP > 1). **(G)** Representative TEM images of LD (indicated by a red asterisk) and lipophagosome, which appeared as double-membrane structure and contained LDs (indicated by a red triangle), in *Abhd11*^KO^ ESCs. Scale bar, 1 μm.

To address the role of ABHD11 in lipid metabolism, we firstly detected the protein level of FASN (Fatty Acid Synthase), which is a key enzyme in *de novo* lipogenesis. Consistent with the mRNA expression, the result showed that the expression of FASN was downregulated in *Abhd11*^KO^ ESCs (Figure 8A). The downregulation of key enzymes in lipid synthesis in *Abhd11*^KO^ ESCs suggested inhibition of *de novo* lipogenesis. Then we analyzed the lipid profiles by LC-MS/MS. Principal component analysis (PCA) of lipid metabolites indicated clear differences among control, *Abhd11*^KO^, and *Abhd11*^OE^ ESCs (Figure 8B). Among the 30 lipid classes detected in ESCs, digalactosyldiacylglycerol and sulfoquinovosyldiacylglycerol (saccharolipids), triglyceride and diglyceride (glycerolipids), phosphatidylethanolamine and phosphatidylglycerol (glycerophospholipids) content showed a significant increase in *Abhd11*^KO^ ESCs while a significant decrease in *Abhd11*^OE^ ESCs (Figures 8C,D and Supplementary Table S6). In addition, KO of *Abhd11* increased the levels of fatty acid and acyl carnitine (fatty acyls) while reduced the levels of ceramides and sphingosine (sphingolipids) (Figures 8C,D and Supplementary Table S6). Volcano plots showed that the lipid species showing the most significant change in content were triglyceride and ceramides in both *Abhd11*^KO^ and *Abhd11*^OE^ ESCs (Figures 8E,F and Supplementary Table S7). The triglyceride content decreased in *Abhd11*^OE^ ESCs while increased in *Abhd11*^KO^ ESCs suggesting that ABHD11 appeared to have substrate preference of triglyceride. Finally, we observed lipid droplets (LD) formation and fusion as well as LD-containing autophagosome (lipophagosome) in *Abhd11*^KO^ ESCs (Figure 8G), suggesting that *Abhd11* KO induced LD growth and lipophagy. Taken together, ABHD11 functions as a key regulator of lipid metabolism in ESCs.

## Discussion

ABHD11 was previously identified as a conserved lipase across species and has a pivotal role in lipid metabolism in Arabidopsis and yeast (Vijayakumar et al., 2016; Arya et al., 2017). In human, *ABHD11* is one of several genes deleted in Williams–Beuren syndrome, a developmental disorder associated with haploinsufficiency of multiple genes (Merla et al., 2002). In addition, ABHD11 is a potential biomarker of lung adenocarcinoma (Wiedl et al., 2011), and is linked to breast cancer malignancy (Heinonen et al., 2015). Despite such reports, however, the actual physiological functions of ABHD11 are unclear. In this study, we characterized the function of ABHD11 in the maintenance of ESCs by both loss- and gain-of-function experiments.

Although ABHD11 has a conserved lipase motif, its endogenous substrates and products are still unknown. In Arabidopsis, the abolishment of ABHD11 activity increases the level of polar lipids in the leaves (Vijayakumar et al., 2016). In yeast, the overexpression of human *ABHD11* or its yeast homolog causes a reduction in the levels of non-polar lipids (Arya et al., 2017). In this study, we observed the opposite effects of OE and KO of ABHD11 on the lipid classes content, such as glycerolipids and glycerophospholipids, supporting its role as a lipid hydrolase. Notably, *Abhd11* KO resulted in the downregulation of enzymes for lipid synthesis, and reduced sphingolipids content in mouse ESCs. It is possible that hydrolyses of substrates by ABHD11 may affect signaling processes contributing to *de novo* lipogenesis in ESCs. In addition, as sphingolipids function as regulators of crosstalk between apoptosis and autophagy (Young et al., 2013), sphingolipids mediates the function of ABHD11 in the expansion and survival of ESCs is an interesting possibility that is worthy of further investigation.

A recent study revealed that regulation of *de novo* lipid synthesis by the lipogenic enzyme, acetyl-CoA carboxylase alpha (ACACA), is critical for ESC pluripotency (Wang et al., 2017). The study also demonstrated that ACACA overexpression and knockdown lead to increased and decreased expression, respectively, of pluripotent genes such as *Oct4* and *Sox2* (Wang et al., 2017). However, we observed that *Abhd11*^KO^ ESCs exhibited decreased lipid synthesis and self-renewal ability, while maintaining a pluripotent state, as well as normal expression of *Oct4* and *Nanog*, suggesting that ABHD11 preserved ESC identity by a mechanism distinct from that of ACACA.

When *Abhd11* expression was suppressed in ESCs, the level of GSK3β phosphorylation significantly increased, indicating a role of ABHD11 in the regulation of GSK3β signaling pathways. The GSK3β is a multifunctional serine/threonine kinase involved in a wide range of cellular processes, ranging from glycogen metabolism to cell cycle regulation and proliferation (Doble and Woodgett, 2003). In ESCs, the inhibition of GSK3β promotes self-renewal through the activation of β-Catenin signaling (Sato et al., 2004; Ying et al., 2008). Thus, enhanced β-Catenin signaling pathway may not be the cause of impaired self-renewal ability in *Abhd11*-depleted ESCs. Notably, RNAseq showed that the expressions of most of the pluripotency regulators were not changed in *Abhd11*^KO^ ESCs (Supplementary Table S2). It is possible that the elevated levels of phosphorylation of GSK3β and nuclear accumulation of β-Catenin were insufficient to increase the expression of the pluripotency genes. Nevertheless, GSK3β inhibition can induce autophagy and cell death (Yang et al., 2010), while GSK3β activation was reported to be either anti-apoptotic or pro-apoptotic (Beurel and Jope, 2006), indicating an impact of GSK3β activity on autophagy and apoptosis. Thus, whether GSK3β inhibition caused by *Abhd11* KO contributes to autophagy or apoptosis is still an open issue.

Notably, our study showed that *Abhd11* overexpression and deletion increased and decreased the expansion of ESCs, respectively, and that *Abhd11* suppression downregulated the expression of metabolic genes related to both lipid biosynthesis and glycolysis. In consideration of the high expression of *ABHD11* in lung cancer (Wiedl et al., 2011; Heinonen et al., 2015) and the key roles of both elevated *de novo* lipid biosynthesis and increased glycolysis in cancer (DeBerardinis and Chandel, 2016), it is crucial to establish whether ABHD11 contributes to malignancy and whether its inhibition can block tumor growth.

In summary, to our knowledge, this study is the first to provide evidence that ABHD11 functions as a key determinant of self-renewal and metabolic homeostasis in ESCs. Our novel discovery explored the broad role of ABHD proteins in lipid metabolism and stem cell biology and has offered new insights into the metabolic regulation of stem cell activity. However, the detailed mechanisms of the suppression of *Abhd11* gene leading to the misexpression of metabolic genes, activation of autophagy, and the disturbances in signal transduction have been left unclarified. Future studies will be required to molecularly dissect the crosstalk among the processes that result from the KO of ABHD11 in ESCs.

## Data Availability Statement

The datasets generated for this study can be found in the GSE142067.

## Ethics Statement

The animal study was reviewed and approved by the Animal Ethical and Experimental Committee of the Third Military Medical University.

## Author Contributions

GL and YR contributed to the conception and design, collection and assembly of the data, data analysis, interpretation, manuscript writing, and financial support. JZ, YT, and JW analyzed the data and interpreted the study, and contributed to the manuscript revision, and final approval of the manuscript. WW, PH, and JX contributed to the administrative support, provision of study material and suggestion. XW, YC, LL, and YY collected and assembled the data, analyzed the data, and interpreted the study. RJ contributed to the conception and design, financial support, data analysis and interpretation, manuscript writing, and final approval of the manuscript. All authors contributed to the article and approved the submitted version.

## Conflict of Interest

The authors declare that the research was conducted in the absence of any commercial or financial relationships that could be construed as a potential conflict of interest.

## Footnotes

1 http://biit.cs.ut.ee/gprofiler/

## References

[B1] Alessi D. R. Andjelkovic M. Caudwell B. Cron P. Morrice N. Cohen P. (1996). Mechanism of activation of protein kinase B by insulin and IGF-1. *EMBO J.* 15 6541–6551. 10.1002/j.1460-2075.1996.tb01045.x 8978681 PMC452479

[B2] Arya M. Srinivasan M. Rajasekharan R. (2017). Human alpha beta hydrolase domain containing protein 11 and its yeast homolog are lipid hydrolases. *Biochem. Biophys. Res. Commun.* 487 875–880. 10.1016/j.bbrc.2017.04.145 28465236

[B3] Beurel E. Jope R. S. (2006). The paradoxical pro- and anti-apoptotic actions of GSK3 in the intrinsic and extrinsic apoptosis signaling pathways. *Prog. Neurobiol.* 79 173–189. 10.1016/j.pneurobio.2006.07.006 16935409 PMC1618798

[B4] Boyer L. A. Lee T. I. Cole M. F. Johnstone S. E. Levine S. S. Zucker J. P. (2005). Core transcriptional regulatory circuitry in human embryonic stem cells. *Cell* 122 947–956. 10.1016/j.cell.2005.08.020 16153702 PMC3006442

[B5] Burdon T. Stracey C. Chambers I. Nichols J. Smith A. (1999). Suppression of SHP-2 and ERK signalling promotes self-renewal of mouse embryonic stem cells. *Dev. Biol.* 210 30–43. 10.1006/dbio.1999.9265 10364425

[B6] Chambers I. Colby D. Robertson M. Nichols J. Lee S. Tweedie S. (2003). Functional expression cloning of Nanog, a pluripotency sustaining factor in embryonic stem cells. *Cell* 113 643–655. 10.1016/s0092-8674(03)00392-1 12787505

[B7] Chu V. T. Weber T. Graf R. Sommermann T. Petsch K. Sack U. (2016). Efficient generation of Rosa26 knock-in mice using CRISPR/Cas9 in C57BL/6 zygotes. *BMC Biotechnol.* 16:4. 10.1186/s12896-016-0234-4 26772810 PMC4715285

[B8] Cong L. Ran F. A. Cox D. Lin S. Barretto R. Habib N. (2013). Multiplex genome engineering using CRISPR/Cas systems. *Science* 339 819–823. 10.1126/science.1231143 23287718 PMC3795411

[B9] Cornacchia D. Zhang C. Zimmer B. Chung S. Y. Fan Y. Soliman M. A. (2019). Lipid deprivation induces a stable, naive-to-primed intermediate state of pluripotency in human PSCs. *Cell Stem Cell* 25 120.e10–136.e10. 10.1016/j.stem.2019.05.001 31155483 PMC7549840

[B10] DeBerardinis R. J. Chandel N. S. (2016). Fundamentals of cancer metabolism. *Sci. Adv.* 2:e1600200. 10.1126/sciadv.1600200 27386546 PMC4928883

[B11] Ding Q. Q. Xia W. Y. Liu J. C. Yang J. Y. Lee D. F. Xia J. H. (2005). Erk associates with and primes GSK-3 beta for its inactivation resulting in upregulation of beta-catenin. *Mol. Cell.* 19 159–170. 10.1016/j.molcel.2005.06.009 16039586

[B12] Doble B. W. Woodgett J. R. (2003). GSK-3: tricks of the trade for a multi-tasking kinase. *J. Cell Sci.* 116(Pt 7), 1175–1186. 10.1242/jcs.00384 12615961 PMC3006448

[B13] Fu Y. Foden J. A. Khayter C. Maeder M. L. Reyon D. Joung J. K. (2013). High-frequency off-target mutagenesis induced by CRISPR-Cas nucleases in human cells. *Nat. Biotechnol.* 31 822–826. 10.1038/nbt.2623 23792628 PMC3773023

[B14] Gu W. Gaeta X. Sahakyan A. Chan A. B. Hong C. S. Kim R. (2016). Glycolytic metabolism plays a functional role in regulating human pluripotent stem cell state. *Cell Stem Cell* 19 476–490. 10.1016/j.stem.2016.08.008 27618217 PMC5055460

[B15] Hassani S. N. Totonchi M. Gourabi H. Scholer H. R. Baharvand H. (2014). Signaling roadmap modulating naive and primed pluripotency. *Stem Cells Dev.* 23 193–208. 10.1089/scd.2013.0368 24147644

[B16] Heinonen H. Lepikhova T. Sahu B. Pehkonen H. Pihlajamaa P. Louhimo R. (2015). Identification of several potential chromatin binding sites of HOXB7 and its downstream target genes in breast cancer. *Int. J. Cancer* 137 2374–2383. 10.1002/ijc.29616 26014856 PMC4744995

[B17] Kim J. (2008). An extended transcriptional network for pluripotency of embryonic stem cells. *Cell* 132 1049–1061. 10.1016/j.cell.2008.02.039 18358816 PMC3837340

[B18] Klionsky D. J. Abdelmohsen K. Abe A. Abedin M. J. Abeliovich H. Arozena A. A. (2016). Guidelines for the use and interpretation of assays for monitoring autophagy (3rd edition). *Autophagy* 12 1–222. 10.1080/15548627.2015.1100356 26799652 PMC4835977

[B19] Kondoh H. Lleonart M. E. Nakashima Y. Yokode M. Tanaka M. Bernard D. (2007). A high glycolytic flux supports the proliferative potential of murine embryonic stem cells. *Antioxid. Redox. Signal.* 9 293–299. 10.1089/ars.2006.1467 17184172

[B20] Lass A. Zimmermann R. Haemmerle G. Riederer M. Schoiswohl G. Schweiger M. (2006). Adipose triglyceridelipase-mediated lipolysis of cellular fat stores is activated by CGI-58 and defective in Chanarin-Dorfman Syndrome. *Cell Metab.* 3 309–319. 10.1016/j.cmet.2006.03.005 16679289

[B21] Li M. Liu G. H. Izpisua Belmonte J. C. (2012). Navigating the epigenetic landscape of pluripotent stem cells. *Nat. Rev. Mol. Cell Biol.* 13 524–535. 10.1038/nrm3393 22820889

[B22] Loh Y.-H. Wu Q. Chew J.-L. Vega V. B. Zhang W. Chen X. (2006). The Oct4 and Nanog transcription network regulates pluripotency in mouse embryonic stem cells. *Nat. Genet.* 38 431–440. 10.1038/ng1760 16518401

[B23] Lord C. C. Thomas G. Brown J. M. (2013). Mammalian alpha beta hydrolase domain (ABHD) proteins: lipid metabolizing enzymes at the interface of cell signaling and energy metabolism. *Biochim. Biophys. Acta* 1831 792–802. 10.1016/j.bbalip.2013.01.002 23328280 PMC4765316

[B24] M N. K. V B. S. C. T. G K. V. B C. S. Guntupalli S. J S. B. (2016). Molecular characterization of human ABHD2 as TAG lipase and ester hydrolase. *Biosci. Rep.* 36:e00358. 10.1042/BSR20160033 27247428 PMC4945992

[B25] Mali P. Yang L. Esvelt K. M. Aach J. Guell M. DiCarlo J. E. (2013). RNA-guided human genome engineering via Cas9. *Science* 339 823–826. 10.1126/science.1232033 23287722 PMC3712628

[B26] Merla G. Ucla C. Guipponi M. Reymond A. (2002). Identification of additional transcripts in the Williams-Beuren syndrome critical region. *Hum. Genet.* 110 429–438. 10.1007/s00439-002-0710-x 12073013

[B27] Moussaieff A. Rouleau M. Kitsberg D. Cohen M. Levy G. Barasch D. (2015). Glycolysis-mediated changes in acetyl-CoA and histone acetylation control the early differentiation of embryonic stem cells. *Cell. Metab.* 21 392–402. 10.1016/j.cmet.2015.02.002 25738455

[B28] Ng H. H. Surani M. A. (2011). The transcriptional and signalling networks of pluripotency. *Nat. Cell Biol.* 13 490–496. 10.1038/ncb0511-490 21540844

[B29] Nichols J. Smith A. (2011). The origin and identity of embryonic stem cells. *Development* 138 3–8. 10.1242/dev.050831 21138972

[B30] Niwa H. Ogawa K. Shimosato D. Adachi K. (2009). A parallel circuit of LIF signalling pathways maintains pluripotency of mouse ES cells. *Nature* 460 118–122. 10.1038/nature08113 19571885

[B31] Obinata D. Takada S. Takayama K. Urano T. Ito A. Ashikari D. (2016). Abhydrolase domain containing 2, an androgen target gene, promotes prostate cancer cell proliferation and migration. *Eur. J. Cancer* 57 39–49. 10.1016/j.ejca.2016.01.002 26854828

[B32] Panopoulos A. D. Yanes O. Ruiz S. Kida Y. S. Diep D. Tautenhahn R. (2012). The metabolome of induced pluripotent stem cells reveals metabolic changes occurring in somatic cell reprogramming. *Cell Res.* 22 168–177. 10.1038/cr.2011.177 22064701 PMC3252494

[B33] Peng Y. Miao H. Wu S. Yang W. Zhang Y. Xie G. (2016). ABHD5 interacts with BECN1 to regulate autophagy and tumorigenesis of colon cancer independent of PNPLA2. *Autophagy* 12 2167–2182. 10.1080/15548627.2016.1217380 27559856 PMC5103361

[B34] Pesce M. Scholer H. R. (2001). Oct-4: gatekeeper in the beginnings of mammalian development. *Stem Cells* 19 271–278. 10.1634/stemcells.19-4-271 11463946

[B35] Reimand J. Isserlin R. Voisin V. Kucera M. Tannus-Lopes C. Rostamianfar A. (2019). Pathway enrichment analysis and visualization of omics data using g:Profiler, GSEA, Cytoscape and EnrichmentMap. *Nat. Protoc.* 14 482–517. 10.1038/s41596-018-0103-9 30664679 PMC6607905

[B36] Ruan Y. He J. Wu W. He P. Tian Y. Xiao L. (2017). Nac1 promotes self-renewal of embryonic stem cells through direct transcriptional regulation of c-Myc. *Oncotarget* 8 47607–47618. 10.18632/oncotarget.17744 28548937 PMC5564591

[B37] Ryall J. G. Cliff T. Dalton S. Sartorelli V. (2015). Metabolic reprogramming of stem cell epigenetics. *Cell Stem Cell* 17 651–662. 10.1016/j.stem.2015.11.012 26637942 PMC4672395

[B38] Sato N. Meijer L. Skaltsounis L. Greengard P. Brivanlou A. H. (2004). Maintenance of pluripotency in human and mouse embryonic stem cells through activation of Wnt signaling by a pharmacological GSK-3-specific inhibitor. *Nat. Med.* 10 55–63. 10.1038/nm979 14702635

[B39] Shiraki N. Shiraki Y. Tsuyama T. Obata F. Miura M. Nagae G. (2014). Methionine metabolism regulates maintenance and differentiation of human pluripotent stem cells. *Cell Metab.* 19 780–794. 10.1016/j.cmet.2014.03.017 24746804

[B40] Shyh-Chang N. Locasale J. W. Lyssiotis C. A. Zheng Y. Teo R. Y. Ratanasirintrawoot S. (2013). Influence of threonine metabolism on S-adenosylmethionine and histone methylation. *Science* 339 222–226. 10.1126/science.1226603 23118012 PMC3652341

[B41] Sperber H. Mathieu J. Wang Y. Ferreccio A. Hesson J. Xu Z. (2015). The metabolome regulates the epigenetic landscape during naive-to-primed human embryonic stem cell transition. *Nat. Cell Biol.* 17 1523–1535. 10.1038/ncb3264 26571212 PMC4662931

[B42] Szulc J. Wiznerowicz M. Sauvain M. O. Trono D. Aebischer P. (2006). A versatile tool for conditional gene expression and knockdown. *Nat. Methods* 3 109–116. 10.1038/nmeth846 16432520

[B43] Teslaa T. Teitell M. A. (2015). Pluripotent stem cell energy metabolism: an update. *EMBO J.* 34 138–153. 10.15252/embj.201490446 25476451 PMC4337063

[B44] Thomas G. Betters J. L. Lord C. C. Brown A. L. Marshall S. Ferguson D. (2013). The serine hydrolase ABHD6 is a critical regulator of the metabolic syndrome. *Cell Rep.* 5 508–520. 10.1016/j.celrep.2013.08.047 24095738 PMC3833083

[B45] Tzelepis K. Koike-Yusa H. De Braekeleer E. Li Y. Metzakopian E. Dovey O. M. (2016). A CRISPR dropout screen identifies genetic vulnerabilities and therapeutic targets in acute myeloid leukemia. *Cell Rep.* 17 1193–1205. 10.1016/j.celrep.2016.09.079 27760321 PMC5081405

[B46] Vijayakumar A. Vijayaraj P. Vijayakumar A. K. Rajasekharan R. (2016). The Arabidopsis ABHD11 mutant accumulates polar lipids in leaves as a consequence of absent acylhydrolase activity. *Plant Physiol.* 170 180–193. 10.1104/pp.15.01615 26589672 PMC4704602

[B47] Wang J. Alexander P. Wu L. Hammer R. Cleaver O. McKnight S. L. (2009). Dependence of mouse embryonic stem cells on threonine catabolism. *Science* 325 435–439. 10.1126/science.1173288 19589965 PMC4373593

[B48] Wang L. Zhang T. Wang L. Cai Y. Zhong X. He X. (2017). Fatty acid synthesis is critical for stem cell pluripotency via promoting mitochondrial fission. *EMBO J.* 36 1330–1347. 10.15252/embj.201695417 28377463 PMC5430220

[B49] Wiedl T. Arni S. Roschitzki B. Grossmann J. Collaud S. Soltermann A. (2011). Activity-based proteomics: identification of ABHD11 and ESD activities as potential biomarkers for human lung adenocarcinoma. *J. Proteomics* 74 1884–1894. 10.1016/j.jprot.2011.04.030 21596165

[B50] Wu J. Belmonte J. C. I. (2016). Stem cells: a renaissance in human biology research. *Cell* 165 1572–1585. 10.1016/j.cell.2016.05.043 27315475

[B51] Yanes O. Clark J. Wong D. M. Patti G. J. Sanchez-Ruiz A. Benton H. P. (2010). Metabolic oxidation regulates embryonic stem cell differentiation. *Nat. Chem. Biol.* 6 411–417. 10.1038/nchembio.364 20436487 PMC2873061

[B52] Yang J. Takahashi Y. Cheng E. Liu J. Terranova P. F. Zhao B. (2010). GSK-3beta promotes cell survival by modulating Bif-1-dependent autophagy and cell death. *J. Cell Sci.* 123(Pt 6), 861–870. 10.1242/jcs.060475 20159967 PMC2831760

[B53] Ying Q. L. Wray J. Nichols J. Batlle-Morera L. Doble B. Woodgett J. (2008). The ground state of embryonic stem cell self-renewal. *Nature* 453 519–523. 10.1038/nature06968 18497825 PMC5328678

[B54] Young M. M. Kester M. Wang H. G. (2013). Sphingolipids: regulators of crosstalk between apoptosis and autophagy. *J. Lipid Res.* 54 5–19. 10.1194/jlr.R031278 23152582 PMC3520539

[B55] Zechner R. Zimmermann R. Eichmann T. O. Kohlwein S. D. Haemmerle G. Lass A. (2012). FAT SIGNALS–lipases and lipolysis in lipid metabolism and signaling. *Cell Metab.* 15 279–291. 10.1016/j.cmet.2011.12.018 22405066 PMC3314979

[B56] Zhang H. Badur M. G. Divakaruni A. S. Parker S. J. Jager C. Hiller K. (2016). Distinct metabolic states can support self-renewal and lipogenesis in human pluripotent stem cells under different culture conditions. *Cell Rep.* 16 1536–1547. 10.1016/j.celrep.2016.06.102 27477285 PMC4981511

[B57] Zhang J. Liu G. Ruan Y. Wang J. Zhao K. Wan Y. (2014). Dax1 and Nanog act in parallel to stabilize mouse embryonic stem cells and induced pluripotency. *Nat. Commun.* 5:5042. 10.1038/ncomms6042 25284313 PMC4205889

[B58] Zhang J. Nuebel E. Daley G. Q. Koehler C. M. Teitell M. A. (2012). Metabolic regulation in pluripotent stem cells during reprogramming and self-renewal. *Cell Stem Cell* 11 589–595. 10.1016/j.stem.2012.10.005 23122286 PMC3492890

[B59] Zhang J. Ratanasirintrawoot S. Chandrasekaran S. Wu Z. Ficarro S. B. Yu C. (2016). LIN28 regulates stem cell metabolism and conversion to primed pluripotency. *Cell Stem Cell* 19 66–80. 10.1016/j.stem.2016.05.009 27320042 PMC13373712

[B60] Zhou Q. Chipperfield H. Melton D. A. Wong W. H. (2007). A gene regulatory network in mouse embryonic stem cells. *Proc. Natl. Acad. Sci. U.S.A.* 104 16438–16443. 10.1073/pnas.0701014104 17940043 PMC2034259

