# Supplementary material for: ABHD11 Is Critical for Embryonic Stem Cell Expansion, Differentiation and Lipid Metabolic Homeostasis
Source: Front Cell Dev Biol. 2020 Jul 7;8:570. doi: 10.3389/fcell.2020.00570 (PMC7358615; doi:10.3389/fcell.2020.00570)
Supplement: Supplementary file 1 [file Image_1.pdf]

Supplementary Material

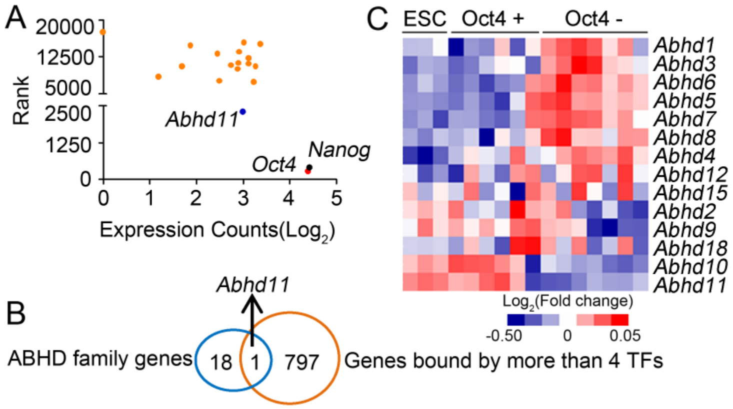

Figure S1. ABHD11 association with ESCs is revealed by bioinformatic analysis. (Related to Figure 1)

(A): Scatter plot showing the ABHD family genes' ranking correlated to ESC viability or maintenance (x-axis) and RNA-seq expression counts in ESCs (y-axis). ABHD family genes except *Abhd11* were presented as orange dots. **(B):** Venn diagram of the overlap between ABHD family genes and common target genes of pluripotency transcription factors (TFs) (Kim, 2008). **(C):** Heatmap representation of gene expression pattern of ABHD family genes in undifferentiated ESCs, OCT4+, and OCT4- cells. Data were from the work of Zhou and colleagues (Zhou et al., 2007).

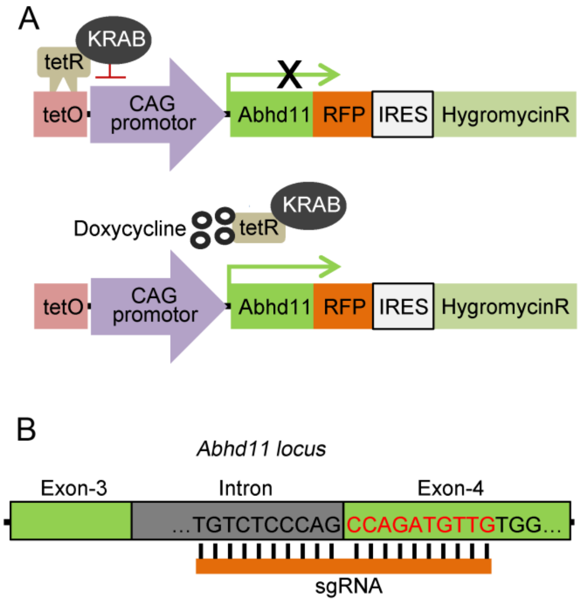

Figure S2. Construction of ESC lines with conditional *Abhd11* expression. (Related to Figure 1)

(A): Schematic representation of the inducible expression of *Abhd11-RFP* by the "tetR-KRAB-tetO" conditional gene expression system. The tetR-KRAB binds to tetracycline repressor operate (*tetO*) and suppresses the expression of *Abhd11-RFP* in the absence of Dox while *Abhd11-RFP* is expressed in the presence of Dox. TetR, tetracycline repressor; KRAB, Krüppel associated box; IRES, internal ribosomal entry site. **(B):** Schematic representation of the design of sgRNA targeting to endogenous *Abhd11*.

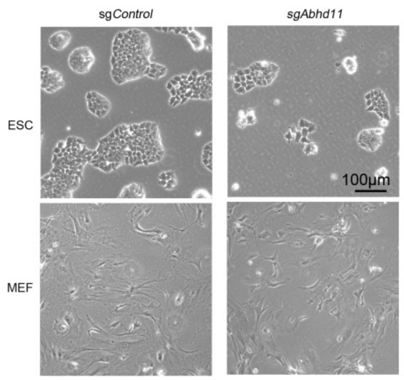

Figure S3. The role of ABHD11 in the expansion of ESCs is cell context dependent. Both ESCs and MEFs were infected with lentivirus expressing Cas9 and sgRNA against *Abhd11(*sg*Abhd11).* Two days after infection, cells were selected with 2 μg/ml purmycin for two days. The resistant cells were replated and cultured for another two days.

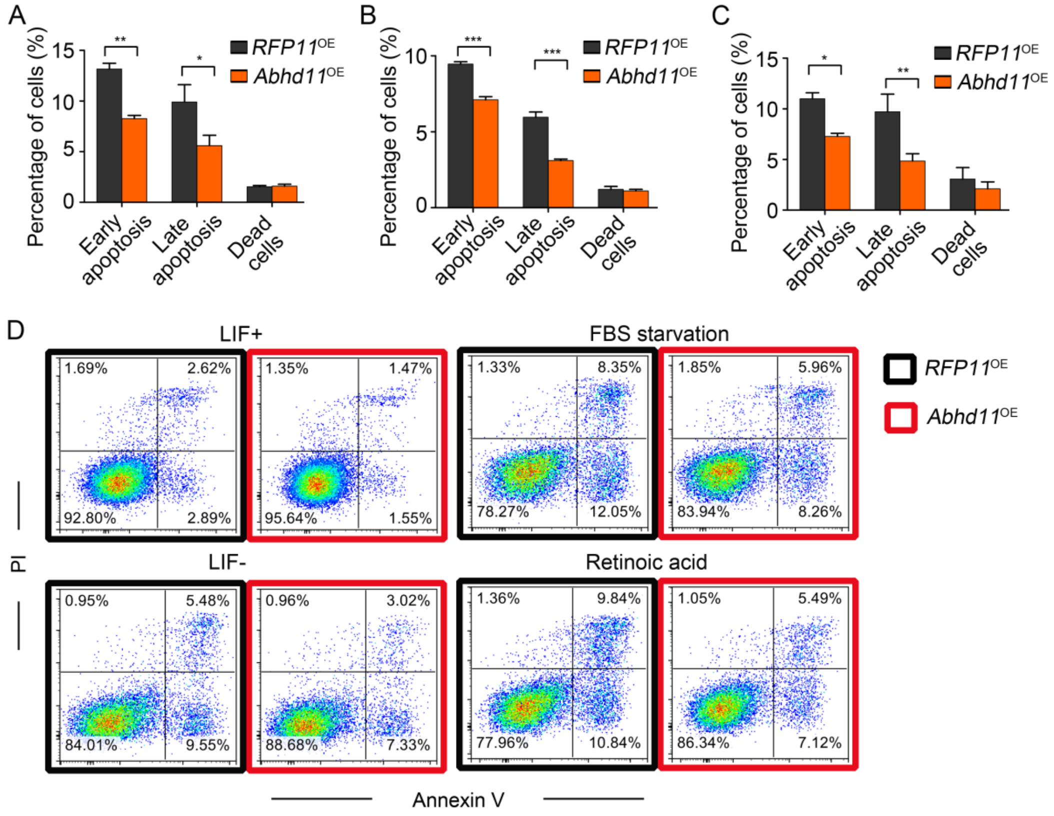

Figure S4. ***Abhd11*** **OE inhibits apoptosis. (Related to Figure 4)**

Analysis of apoptosis in *RFP*^{OE} ESCs and *Abhd11*^{OE} ESCs in different culture conditions by FACS with Annexin-V and PI staining. **(A-C):** The fraction of the indicated cells labeled for early apoptosis (Annexin V+PI-), late apoptosis (Annexin V+PI+), and dead cells (Annexin V-PI+). **(A):** Cells were cultured in ESC maintenance medium for 24 hours and then changed to medium without serum; **(B):** Cells were induced to differentiation in the absence of LIF; **(C):** Cells were induced to differentiation by addition of 1×10^{-7} M retinoic acid. Data in **A**, **B**, and **C** were represented as mean ± s.d.; $n = 3$. $*p < 0.05$, $**p < 0.01$, $***p < 0.001$. **(D):** Apoptosis of the indicated cells was measured by FACS with Annexin-V and PI staining. One of three experiments with similar results was shown.

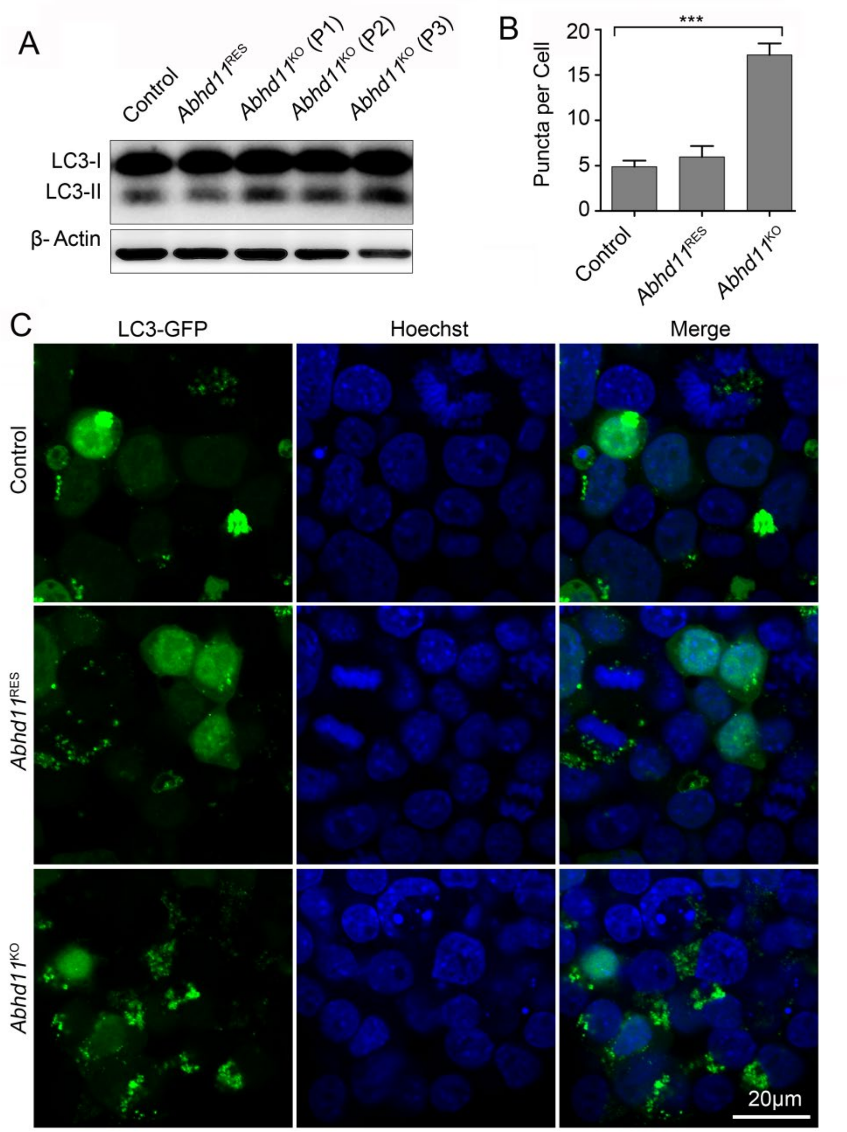

Figure S5. ***Abhd11*** **KO induces autophagy. (Related to Figure 4)**

(A): Western blot analysis of LC3-I and LC3-II expression level in the indicated cells during passage (P) with anti- LC3 antibody. Cells were cultured in the presence of 25μm chloroquine. **(B):** Quantification of GFP-LC3 puncta in the indicated cells. Data were represented as mean ± s.d. from three independent experiments and at least 50 cells in each experiment were counted. ***$p < 0.001$. Cells were cultured in the presence of 25μm chloroquine. **(C):** Confocal fluorescence microscopy images showing the subcellular localization of GFP-LC3 in the indicated cells. Cells were cultured in the presence of 25μm chloroquine.

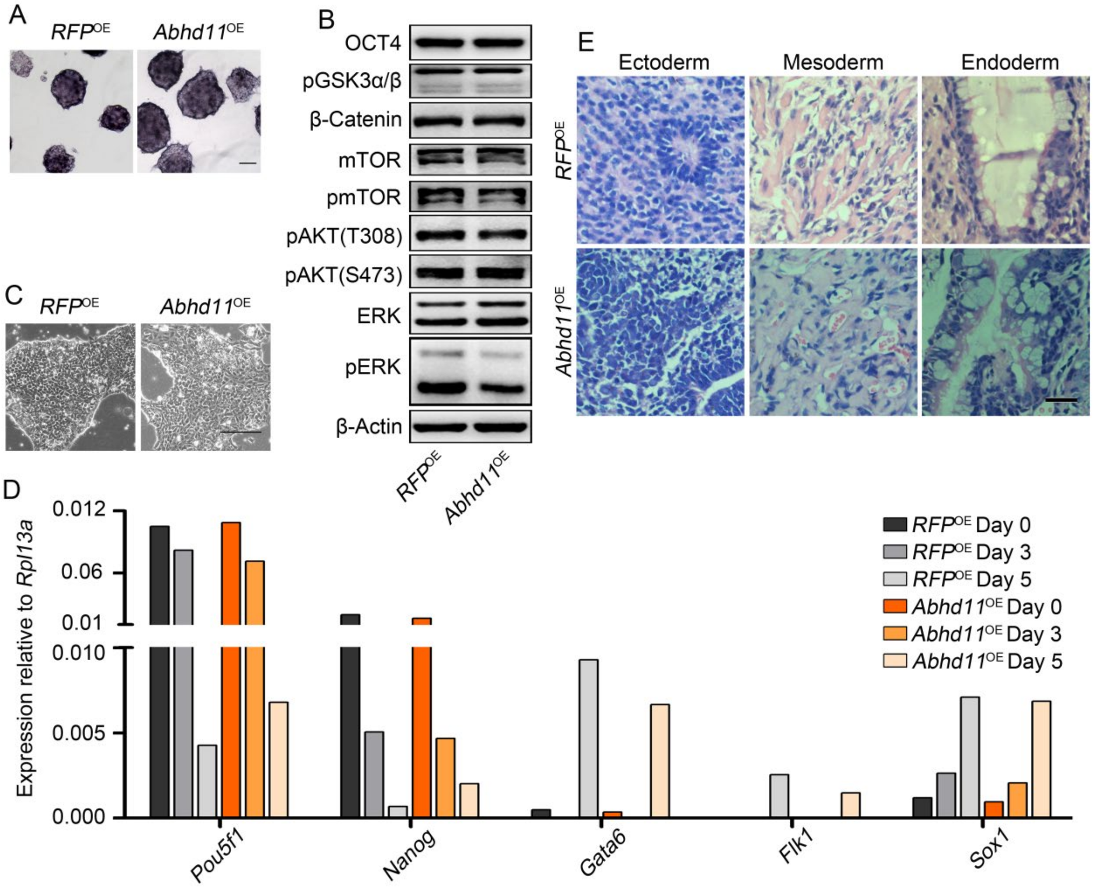

Figure S6. ABHD11 OE neither altered pluripotency nor blocked diffefentiation of ESCs.

(A): Alkaline phosphatase staining of colonies formed by the indicated cells. Scale bar, 100 µm. **(B):** Western blot analysis using antibodies as indicated. **(C):** Representative images of the indicated cells cultured for 4 days in the absence of LIF. **(D):** qRT-PCR analysis of expression levels of pluripotency and germ layer marker during the course of EB formation. The EBs were collected at Day0, 3, 5 during the course of EB formation. All data were normalized to *Rpl13a*. **(E):** Teratoma sections were stained with hematoxylin/eosin.

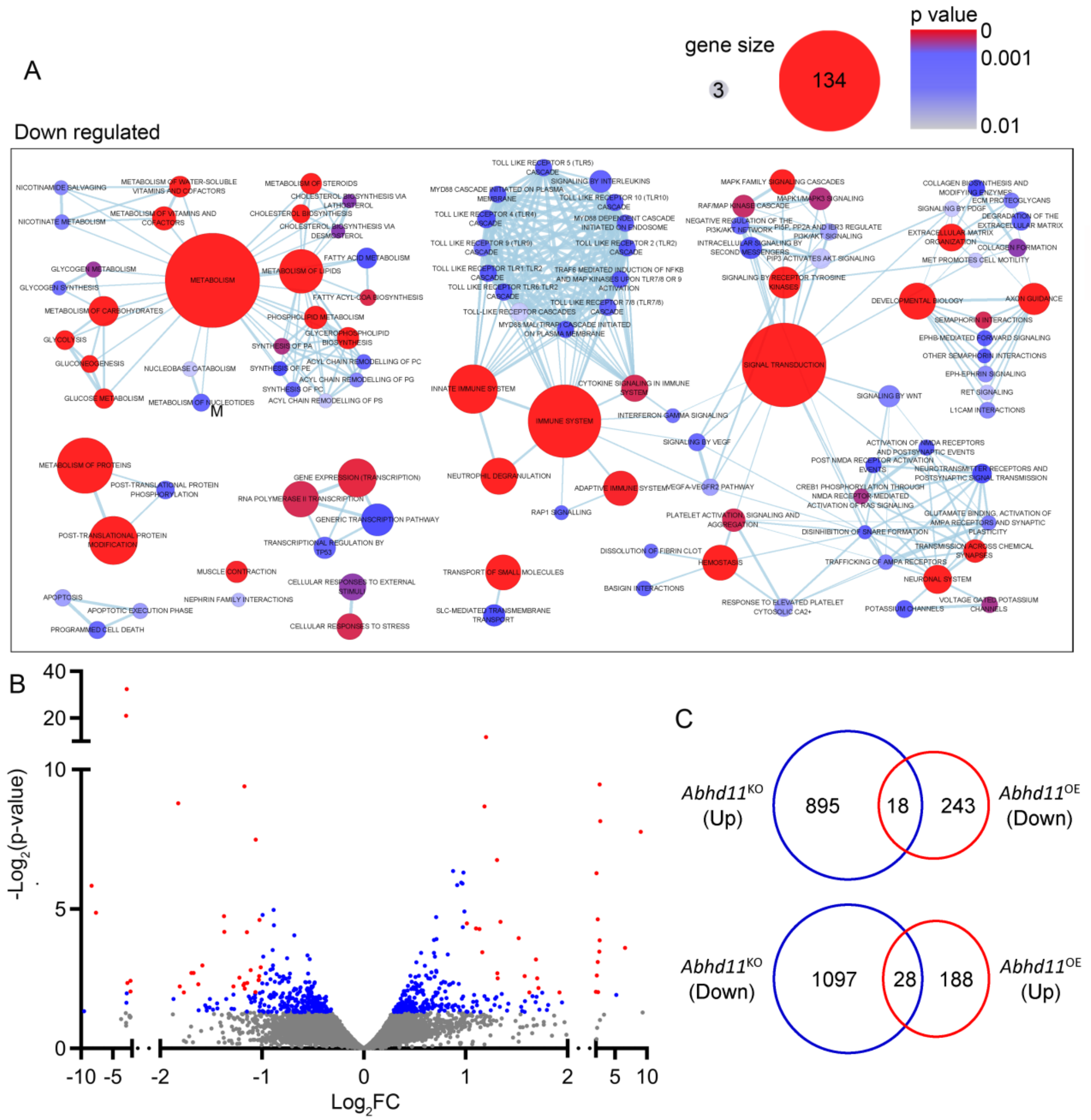

Figure S7. Enrichment map networks of reactome terms corresponding to DEGs in *Abhd11*^{KO} ESCs. (Related to Figure 6)

(A): The reactome terms enrichment analysis for down-regulated genes in *Abhd11*^{KO} ESCs were analysed using the web resource (http://biit.cs.ut.ee/gprofiler/gost) and visualized by the cytoscape plug-in: Enrichment Map. Node size represents the gene-set size and node color represents enrichment significance (as indicated). **(B):** Volcano plot of DEGs in *Abhd11*^{OE} ESCs versus control ESCs. FC, fold change. **(C):** Venn diagram of the overlap between DEGs in *Abhd11*^{KO} and DEGs in *Abhd11*^{OE} ESCs.

Supplemental tables

Table S1. ESC transcriptome data measured by RNA seq.
Table S2. List of DEGs.
Table S3. The GO functional enrichment analysis for DEGs upon *Abhd11* depletion.
Table S4. List of primers used for PCR.
Table S5. List of antibodies used for western blots.
Table S6. The lipid intensity of lipid classes.
Table S7. The lipid intensity of lipid species.

Supplemental references

Kim, J. (2008). An Extended Transcriptional Network for Pluripotency of Embryonic Stem Cells. Cell 132, 1049-1061.

Zhou, Q., Chipperfield, H., Melton, D.A., and Wong, W.H. (2007). A gene regulatory network in mouse embryonic stem cells. Proceedings of the National Academy of Sciences 104, 16438-16443.
